# Ilizarov versus ORIF for Open AO Type-C Pilon Fractures

**DOI:** 10.12669/pjms.40.10.8941

**Published:** 2024-11

**Authors:** Saeed A. Shaikh, Dr Malik Osama Tanveer, Muhammad Tahir, Nadeem Ahmed

**Affiliations:** 1 Dr. Saeed A Shaikh, FCPS, Department of Orthopaedics, Surgical Building, Jinnah Postgraduate Medical Centre, Rafiqui Shaheed Road, Karachi, Pakistan; 2 Dr Malik Osama Tanweer, FCPS. Consultant Orthopaedics, Faculty of Surgery, South City Institute of Physical Therapy and Rehabilitation, Karachi, Pakistan; 3 Mr. Muhammad Tahir, MRCS Eng, FCPS, Department of Orthopaedics, Surgical Building, Jinnah Postgraduate Medical Centre, Rafiqui Shaheed Road, Karachi, Pakistan; 4 Dr. Nadeem Ahmed, FCPS, Department of Orthopaedics, Surgical Building, Jinnah Postgraduate Medical Centre, Rafiqui Shaheed Road, Karachi, Pakistan

**Keywords:** Open fracture, Pilon fracture, Arthritis, AOFAS-Ankle Hindfoot Score, Pain

## Abstract

**Objective:**

This study aimed to evaluate the clinical effectiveness of ORIF versus Ilizarov for the management of Type C closed pilon fractures of the distal tibia at 12 months follow up.

**Methods:**

This retrospective cross sectional study was conducted at Jinnah Postgraduate Medical Center (JPMC) between 29^th^ May 2015 and 27^th^ November 2019 that included patients 18 years and older diagnosed with open AO type C pilon fractures. The primary outcome was the patient-reported Disability Rating Index (DRI) months. While the secondary outcomes were quality of life assessment using the patient satisfaction form (SF-12) and AOFAS-Ankle Hindfoot Score. Radiographs were assessed for fracture healing, time to healing, and malalignment.

**Results:**

Fifteen patients underwent ORIF, while 26 patients were treated with Ilizarov, there was no statistically significant difference in DRI scores at 12 months between the two groups. In terms of clinical outcomes, both groups had comparable results throughout the follow-up period. The number of unplanned surgical procedures was not statistically significant (p=0.73), 26.92% (n=7) in the Ilizarov as compared to 33.33% (n=5) in the ORIF group.

**Conclusion:**

Among patients with an acute, displaced, intra-articular fracture of the distal tibia, neither external fixation nor locking plate fixation resulted in superior disability status at 12 months. Patient factors may need to be considered in deciding the optimal approach.

## INTRODUCTION

Pilon fractures are complicated ankles caused by high-energy trauma such as road traffic injuries or falls from great heights. Axial loading with a rotational element is the mechanism of injury. Approximately 5–7% of all tibial fractures are pilon fractures.^1^ Patient variables such as complicated fracture patterns (such as AO Type-C), limited soft-tissue coverage, poor vascular supply, smoking habits, and comorbidities might make pilon fracture care challenging. These characteristics complicate pilon fracture surgery and can lead to significant consequences, such as deep infections, osteomyelitis, delayed or nonunion, and post-traumatic arthritis.^2^ These complications can potentially lead to additional surgical procedures to treat infections, secondary arthritis, and amputations, resulting in long-term impairment.^3^

Pilon fractures of the tibia have been treated using various techniques, although the ideal therapy remains debatable. In Europe, these patients are traditionally treated with open reduction and internal fixation (ORIF) of the tibia with bone grafting as necessary to restore the distal fibula length and articular surface.^4^ In contrast, several North American trauma centers have treated these patients with immediate external fixation and delayed internal fixation of the articular surface with minimal incisions, leaving the external fixator in place until bone union. As reported in the literature, this approach for plafond fractures yields favorable outcomes with low complication rates.^5^

Recently, however, with the Ilizarov apparatus, it has become possible to treat such patients with a single-stage treatment because reduction is less invasive, with minimal soft-tissue exposure and blood loss.^6^,^7^ If necessary, Ilizarov also permits alignment modification and compression/distraction during and after surgery. Additionally, the fixation is sufficiently stable to permit early weight bearing.

Surgeons at our institute disagree on the optimal treatment for severe pilon fractures. Therefore, the purpose of this retrospective study was to determine whether ORIF or external fixation with an Ilizarov device results in (1) superior patient-reported Disability Rating Index scores and (2) higher patient-reported outcome scores, including patient satisfaction form (SF-12) and AOFAS-hindfoot and ankle scores, when treating closed Type C pilon fractures. (3) an increased likelihood of union after nine months or (4) an increase in unscheduled reoperations.

## METHODS

This retrospective cross sectional study was conducted at Jinnah Postgraduate Medical Center (JPMC) between 29^th^ May 2015 and 27^th^ November 2019. Patients aged 18 years and older who underwent surgical repair for open intra-articular pilon with a minimum of 12-18 months of clinic follow-up were evaluated retrospectively from a prospectively collected observational cohort. The surgeries were performed at a Level-1 trauma center and the private practices of the participating consultants. The operating surgeons were orthopaedic consultants and were AO trauma certified, each having an 80% dedicated practice to orthopaedic trauma and adequate experience in dealing with complex trauma, each having a minimum of eight years of consultant experience. We identified 16 patients who underwent ORIF, while 25 were treated with Ilizarov. We excluded patients with metabolic bone disease, pathological fractures, and those unable to understand and retain the information due to mental illness, as this would result in recall bias.

### Ethical Approval:

The study was approved by the institutional review board of Jinnah Postgraduate Medical Centre, Karachi with reference number (NO. F 2-81/ GENL-2022/167/JPMC, dated April 18, 2022). An informed consent was obtained from all participants.

The initial orthopaedic management of open pilon fractures after the advanced trauma life support was debridement, wound lavage, analgesia, reduction, and application of an above-knee splint in the orthopaedic bay. Since there was an equipoise among our trauma consultants on how to manage these fractures, they were treated according to the on-call consultant’s protocol for managing these fractures. The consultant who favored ORIF had a protocol for assessing soft tissues for swelling and blisters during the next day’s ward rounds. If the condition of the soft tissues was permitted, and the patient was fit for anesthesia, patients were operated within 48 hours of presentation; otherwise, a calcaneal traction pin was passed, and the injured extremity was elevated on a Bohler-Braun frame to allow swelling and blisters to subside and then proceed for surgical fixation.

The median (range) time from injury to surgical intervention was five days (range, 3–7 days). All patients received preoperative antibiotics, which were continued for 48–72 hours. Deep venous thromboprophylaxis (DVT) was administered according to the hospital protocol. ORIF was performed under general or spinal anesthesia.

During ORIF, the locking plate was inserted using a minimally invasive plate osteosynthesis technique that involved dissection of the distal tibia and sliding of the plate submuscularly. The details of the surgical approach, reduction techniques, implant choice, fixation methods, and supplementary devices or techniques were left to the surgeon’s judgment. Fracture reduction was achieved by opening the fracture site and achieving reduction under vision and C-arm guidance. The only stipulation was that fixed-angle screws were applied to the distal holes of the implant, which is standard practice for all distal tibial locking plates. An autograft was used if shortening was anticipated or to promote union, depending on the surgeon’s intraoperative decision.

An Ilizarov external fixator (SK Surgicals, Karachi, Pakistan) was used for pilon fracture stabilization. The surgical technique, construction of the frame including rings, wires, and half pins, and use of bone graft were left to the discretion of the surgeon. The frames were manually constructed. Fracture reduction was achieved using a closed method under fluoroscopic guidance. Limited internal fixation with an Ilizarov external fixator was not practiced in our hospitals; therefore, it was not used. The ankle joint was spanned with a fixator in all patients. All Ilizarov frames have footplates. Foot plate removal was performed at the discretion of the surgeon. The frames were compressed along with corticotomy in patients with nonunion. In patients with delayed healing or nonunion, the Ilizarov frames remain in situ for the duration of fracture healing.

There is a unified postoperative care protocol, regardless of the fixation method. DVT prophylaxis was initiated on the first postoperative day according to the institutional protocol. Intravenous and oral analgesics, along with intravenous antibiotics, were administered. Ankle and knee exercises were initiated on the second postoperative day by a physiotherapist. Partial weight-bearing was allowed when radiologic evidence of early union was observed. Full weight bearing was allowed when there was radiologic evidence of union with no pain at the fracture site. All Ilizarov fixators were removed under local anesthesia in the clinic, and they did not count towards unplanned procedures.

All trauma patients in our department were followed up for a minimum of 12 months with the operating surgeon and the physiotherapist in the clinic and thereafter on an annual basis with the physiotherapist, where patient-reported outcomes are routinely collected for audit purposes with the consent of the patients keeping their identity anonymous.

For all orthopaedic injuries, the primary outcome in our institute is the patient-reported Disability Rating Index.^8^ Disability Rating Index scores range from 0 to 100 points, with 0 indicating normal function and 100 indicating complete disability. The Disability Rating Index is a validated patient-reported outcome measure. It has been proven to be a potent, practical clinical, and research instrument with good responsiveness and acceptability for the assessment of disability caused by lower limb impairment.^9^

The secondary outcome assessed at follow-up was patient satisfaction using the SF-12 patient satisfaction Form.^10^ Depending upon the specific injury, a limb-specific outcome measure was used in the department for this case, which was the AOFAS-hindfoot and ankle score.^11^ SF-12 scores range from to 0-100 points, with 0 being the lowest level of health of an individual. The AOFAS-hindfoot and ankle score, devised for patients with hindfoot and ankle pathologies, ranges from 0 to 100 points, with 0 indicating totally impaired function and 100 indicating completely unimpaired function.

Wound dehiscence, drainage, or cellulitis were described as superficial infections that were treated conservatively with oral antibiotics and topical wound care. Deep infections were defined as those that required surgical debridement and lavage. Patients who required a secondary intervention to establish union were categorized as non-union patients. Unplanned operations were recorded for both groups. Malunion was defined as an angulation of > 5º in either plane or a shortening of > 1 cm. On postoperative radiographs, malunion was defined as 5º angulation in the coronal plane, 10º angulation in the sagittal plane, or 2mm of articular step-off. On standing anteroposterior, lateral, and mortise radiographs of the ankle, the angle generated by the intersection of the subchondral line of the plafond and a line drawn along the center of the tibial shaft was measured to determine the alignment. The normal was 90º, and variations of 5º were recorded as varus, valgus, anterior, or posterior angulation.

We evaluated and reported early- and mid-term impairment at three, six, and nine months. All continuous tests were two-sided, and the significance threshold was set at 95%. Chi-square and Fisher exact tests were used for categorical variables. Using SPSS 22.0.1, primary and secondary outcomes were analyzed using SPSS (IBM Corp). Statistical significance was set at P < 0. 05.

## RESULTS

We found no differences between the Ilizarov and ORIF groups in terms of Disability Rating Index, SF-12, or AOFAS-hindfoot and ankle scores at 12 months of follow-up. In addition, there was no difference between the groups in the proportion of patients who experienced nonunion ORIF versus Ilizarov. More unplanned procedures were performed in the Ilizarov group than in the ORIF group.

The mean age of the 15 enrolled patients (86.7% males, n=13) in the plating group was 31.8±4.73 years whereas 88.5% males n=23/26 in Ilizarov group had a mean age of 44±9.16 years, respectively. The mean weight of patients who underwent ORIF procedure was 87.47±7.80 kg whereas patients with external fixators (EF) had a lower mean of 80.69±12.19 kg. Due to this variance, there was a significant mean difference seen in the BMIs score (p=0.02) with a mean BMI of 31.18±1.27 kg/m2 in the ORIF group and a mean BMI of 28.86±3.76 kg/m2 in the Ilizarov group. Sequentially, a significant difference was found when patients from both groups were compared on more or less than 30 kg/m2 criteria (p =0.04), with 61.5% of patients in the EF group having less than 30 kg/m2 and 86.7% having more than 30 kg/m2.

According to the AO/OTA classification, in the ORIF group, 46.7% of the patients had C1 fracture type, and C2 and C3 fracture types were in the same range (26.7%). C1 fracture type was less common (19.2%) in the Ilizarov group. According to the Gustilo-Anderson Grading, only one patient (6.7%) was in Grade-3B of the ORIF group, whereas four patients (15.4%) were of the same grade in the Ilizarov group. Overall, no significant difference was found in ASA grade, AO/OTA fracture type, Gustilo-Anderson Grading, and polytrauma status between the groups. The details are presented in Table-I.

**Table-I:** Comparison of Patient Reported Outcomes.

Outcome	ORIF (15)	Ilizarov Group (26)	P-value
***Primary outcome***			
DRI at 12 months	39.82 ± 6.26	36.55±6.98	0.14
***Secondary outcomes***			
DRI scores at 3 months	45.89 ±4.69	48.69 ±8.36	0.24
DRI scores at 6 months	42.64±5.27	40.95±7.633	0.45
DRI scores at 9 months	39.60±6.90	40.69±5.61	0.58
***SF-12 Physical Component Scale***			
At 3 months	41.38±7.82	39.76±7.32	0.50
At 6 months	40.41±9.97	38.66±9.03	0.56
At 9 months	38.27±6.09	37.88±6.20	0.84
At 12 months	42.08±7.75	43.31±6.51	0.59
***SF-12 Mental Component Scale***			
At 3 months	39.78±6.40	36.58±7.63	0.17
At 6 months	42.83±7.59	40.00±8.47	0.29
At 9 months	41.98±6.02	41.91±9.54	0.62
At 12 months	39.50±4.76	38.54±6.58	0.98
***AOFAS Ankle-Hindfoot Scale***			
At 3 months	41.70±7.97	44.52±13.52	0.46
At 6 months	45.97±10.33	48.26±7.28	0.41
At 9 months	57.14±4.66	53.62±7.61	0.11
At 12 months	60.98±11.49	63.93±10.58	0.41
Time to radiographic union (weeks)	16.00 ±1.85	15.92± 1.74	0.89

DRI: Disability rating index; AOFAS Ankle-Hindfoot Scale: American Orthopaedic Foot and Ankle Society: Ankle-Hindfoot Rating System.

The primary and secondary outcomes of these procedures are presented in Table-I. Regarding the complications in both groups, complex regional pain syndrome (CRPS), malunion and amputation were seen in one (6.7%) patient each that had an ORIF procedure. Four patients (15.4%) had malunion in the Ilizarov group, whereas nonunion, superficial, deep infection, and pin tract infection were experienced by two patients (7.7%) each. Similar to the ORIF group, one patient (3.8%) experienced an amputation complication during the EF procedure. This information is further presented in Table-II.

**Table-II:** Complications in both groups

Complications	ORIF (15)	Ilizarov (26)	p-value
CRPS	1 (6.7%)	0	0.36
DVT	0	0	N/A
Secondary arthritis	3	4	0.72
Malunion	1(6.7%)	4(15.4%)	0.63
Nonunion	0	2	0.52
superficial infection	0	2(7.7%)	0.52
Pin tract Infection		2 (7.7%)	N/A
Deep infection	0	2 (7.7%)	0.52
Unplanned surgical procedures			0.000
Amputations	1(6.7%)	1(3.8%)	1.00

CRPS: Complex Regional Pain Syndrome, DVT: Deep Venous Thrombosis.

## DISCUSSION

This trial aimed to address this question and determine which of the two surgical options, Ilizarov external fixation or ORIF, would lead to superior patient-reported outcomes and fewer complications. Our results showed no clinically important differences in outcome scores when treating open Type-C pilon fractures with ORIF or Ilizarov; however, they showed more unplanned (and planned) reoperations among patients treated with the Ilizarov approach. Therefore, we recommend ORIF for patients with these injuries, unless there is a compelling reason to use the Ilizarov approach in a particular circumstance (such as soft tissue injuries precluding internal fixation).

Some studies have reported complications in patients with external fixators (EF). For instance, infection at the pin site (20%) and a 100% success rate for healing fractures were minor problems that Bone et al. discovered in patients receiving EF.^12^ Four pin infections (15%) and one deep infection (8%) were documented by Tornetta.^13^ This closely mirrors our result of 7.7% deep infections in EF-treated patients.

According to a meta-analysis, the EF group had a noticeably higher risk of nonunion than the ORIF cohort, with the EF cohort having a higher incidence of superficial infection than the ORIF group (9.3%), although there was no difference between the two cohorts in terms of deep infections.^14^ The current study showed results of superficial infection, consistent with the above-mentioned meta-analysis (7.7% vs. 9.3%). The use of EF for an extended period is linked to pin-track infections, and the possibility of septic arthritis is increased by the presence of Schanz pins or wires close to the joint.^15^ Kumar et al observed that all the fractures that experienced delayed union—11 proximal and four distal—were able to heal on their own.^16^

In contrast, Lim et al. definitively managed open pilon fractures with fine wire fixation and observed superficial infection in nine cases and deep infection in one case.^17^ Infections occurred, and delayed union occurred in four situations, all of which were correlated to a high Gustilo-Anderson grade. Hu et al. investigated Gustilo I and II pilon fractures treated via ORIF using a lateral approach did not report significant cases of infection, skin necrosis, or symptomatic implants.^18^ Following CEF, pin tract infections occurred in 13 of 59 patients (22%) in a study conducted in the UK, which is higher than the present study.^19^

In contrast, according to several researchers, ORIF causes serious soft tissue problems including deep infections.^20^ Olson et al. reported a 17% rate of deep infection in the ORIF group from a sample of 401 patients.^21^ Open fracture superficial infection rates ranged from 6% to 8% in studies of a smaller size and from 8% to 28% and 43% in larger studies, respectively.^22^

According to the most recent ORIF literature, the overall infection percentages (superficial: open, 54%; closed, 21%; deep: open, 10%; closed, 7%) were comparable.^23^ Post traumatic arthritis (16%) and nonunion (24%) were the most frequent sequelae for open fractures, while superficial infection (21%) and posttraumatic arthritis (24%) were the most frequent for closed fractures.^24^ In another study in Italy, patients had fractures of AO Type-43C7. Three Gustilo Type-IIIA, seven Type-III B, and four Type-III C injuries were reported.^24^ Delays in union were noted in six individuals (43%).^25^

The primary union percentage was 58%, according to one research.^23^ Approximately 10 months passed before six patients (42%) with delayed union recovered.^25^ The deep infection that manifested in 67% of cases may have contributed to the relatively high prevalence of delayed union.^23^ The superficial infection affected seven out of 76 (9.2%) patients who received ORIF; deep infection affected just two patients (2.6%) and required a formal debridement; no flap was necessary.^20^ Treatment using the Ilizarov external fixator technique in conjunction with minimal ORIF was performed in one of the studies.^25^ Following bone grafting, three patients experienced pin-site infections and one patient experienced a profound infection.^25^ Both ORIF and EF procedures have complications, but the latest literature supports the narrative presented in this study that until there is a good reason to compel the EF procedure, it is better to perform the ORIF procedure depending on the injury.

### Limitations & strengths of study:

This study had several limitations. First, the study was retrospective and exposed to potential recall bias. In addition, there were no clinical data regarding the baseline function of the participants that could be compared to the clinical outcomes after the intervention. This limitation was minimized by excluding patients with previously impaired ankle function.

The purpose of this study was to compare these two fixation methods. Fixation can be achieved by different methods/approaches depending on the fracture pattern and surgical expertise. We note that no two fractures are the same, and every fracture requires a certain amount of customization of the approach depending on the fracture pattern and soft tissue condition. Therefore, the details of the surgical intervention were left to the discretion of the operating surgeons, which may have resulted in variability in terms of the treatments received by the ORIF group. All operating surgeons were appropriately trained in their field and had extensive experience in managing orthopaedic trauma.

Similarly, physical therapists were allowed some discretion, although the main approaches were comparable. We do not believe that either of these limitations, which reflect both trauma care and physical therapy in real-world conditions, undermines the main conclusions of our study. Therefore, the substantially increased risk of reoperation after Ilizarov external fixation should cause clinicians to approach this technique with great caution and only when ORIF seems inappropriate, such as if a patient has compromised soft tissue coverage, a severe open fracture, or severe patient comorbid factors that could increase the risk of complications with external fixation. Considering this, we recommend ORIF for the management of most Type-C pilon fractures as most surgeons are familiar with the technique,

## CONCLUSION

Neither ORIF nor the Ilizarov method of skeletal stabilization yielded superior outcomes in disability status and clinical and health-related outcome metrics at the one year’s follow-up after complete intra-articular pilon fractures. The proportion of patients with unplanned surgical interventions was higher in the Ilizarov group.

### Author`s Contribution:

**SAS:** Recruited patients and performed Ilizarov, reviewed the initial and final draft and final approval of the study and he is responsible and accountable for the accuracy or integrity of the work

**MOT:** Prepared the second draft, interpretation bibliography, collection of PROMs, final approval of manuscript

**MT:** Conception of study, initial manuscript writing, results, interpretation bibliography, final approval of manuscript

**NA:** Supervision of study and performed plating, validation of study, final approval of the manuscript.
